# Serotype IV Sequence Type 468 Group B *Streptococcus* Neonatal Invasive Disease, Minnesota, USA

**DOI:** 10.3201/eid2211.152031

**Published:** 2016-11

**Authors:** Sarah Teatero, Patricia Ferrieri, Nahuel Fittipaldi

**Affiliations:** Public Health Ontario, Toronto, Ontario, Canada (S. Teatero, N. Fittipaldi);; University of Minnesota Medical School, Minneapolis, Minnesota, USA (P. Ferrieri); University of Toronto, Toronto (N. Fittipaldi)

**Keywords:** Bacteria, group B *Streptococcus*, serotype IV, whole-genome sequencing, neonatal invasive disease, recombination, Minnesota,

## Abstract

To further understand the emergence of serotype IV group B *Streptococcus* (GBS) invasive disease, we used whole-genome sequencing to characterize 3 sequence type 468 strains isolated from neonates in Minnesota, USA. We found that strains of tetracycline-resistant sequence type 468 GBS have acquired virulence genes from a putative clonal complex 17 GBS donor by recombination.

Group B *Streptococcus* (GBS) is an opportunistic pathogen responsible for infections in neonates and adults; infection results in substantial morbidity and mortality worldwide (*1*). Ten serotypes (Ia, Ib, II–IX) have been described on the basis of their capsular polysaccharide antigen (*2*). A widely used multilocus sequence typing (MLST) scheme enables discrimination of GBS strains in >700 sequence types (STs), which are grouped in a few clonal complexes (CCs) (*3*).

We recently reported increased frequency of isolation of both carriage and invasive serotype IV GBS in Minnesota, USA, and increased frequency of isolation of serotype IV strains causing invasive disease in 3 Canadian provinces (*4*–*7*). We also showed that emerging serotype IV GBS organisms are genetically heterogeneous. Although most serotype IV strains belonged to ST459 (CC1) or ST452 (CC23), the population also comprised ST3, ST196, ST710, ST711, ST291, ST682, and ST468 isolates, the latter a single-locus variant of ST452 (*4*–*7*). Little is known about several of these less common serotype IV STs causing human disease, including their genomic makeup and antimicrobial drug resistance profiles. Also unknown are the molecular mechanisms underlying their emergence.

Homologous recombination occurs frequently in GBS and can involve vast areas of the genome in some lineages (*8*,*9*). Although MLST does not permit detection of recombination events with accuracy, the use of now relatively inexpensive next-generation whole-genome sequencing (WGS) has enabled the study of recombination in GBS at the population level (*4*,*9*,*10*). We used WGS analysis of 3 ST468 isolates recovered from neonates in Minnesota to test the hypothesis that recombination is a main driver of genetic diversity among serotype IV GBS.

## The Study

We studied 3 ST468 strains, 2 isolated in 2007 and 1 in 2010 from neonates with early-onset or late-onset GBS disease (Table). We prepared genomic DNA by using a QIAGEN DNA MiniKit (QIAGEN, Toronto, ON, Canada) and genomic libraries by using Nextera XT kits (Illumina, San Diego, CA, USA). We sequenced the libraries as paired-end reads (150 bp + 150 bp) by using a MiSeq instrument (Illumina). We determined pili content in silico by aligning short-read WGS data with the sequences of pilus island (PI) 1, PI-2a, and PI-2b and using MOSAIK (*5*). The 3 ST468 strains possessed PI-2b only.

**Table:** Strains of neonatal invasive group B *Streptococcus* disease used in geneticdiversity study, Minnesota, USA*

Isolate no.	Year cultured	Culture source	Patient age, d	SNPs to NGBS572	SRA accession no.
PF-10	2010	Blood	0	766	SRR4101407
PF-17	2007	Blood	2	706	SRR4101408
PF-18	2007	Blood	9	704	SRR4101409

*All isolates were serotype IV (*6*). SNP, single-nucleotide polymorphism; SRA, Sequence Read Archive.

**Figure 1:**
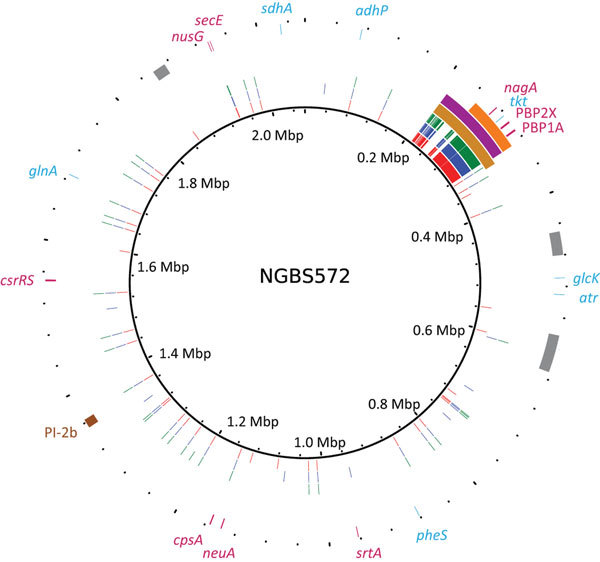
Genome analysis that identified recombination in sequence type (ST) 468 strains of group B *Streptococcus*. Plotting of polymorphisms identified in ST468 strains (PF-10, PF-17, and PF-18 in red, blue, and green, respectively) relative to the genome of the ST452 strain NGBS572 shows areas of densely clustered polymorphisms between positions 210,000 and 320,000 bp. Genome landmarks, such as mobile genetic elements (gray), multilocus sequence typing genes (light blue), pilus (brown), and virulence genes (dark pink), are marked in the outer circle. Precise areas of recombination in ST468 strains defined by BRAT NextGen (*12*) are depicted in gold (PF-10), purple (PF-17), and orange (PF-18). Mbp, megabase pairs.

We next aligned the short reads to the genome of serotype IV ST452 strain NGBS572 (GenBank accession no. CP007632.1) and identified polymorphisms by using a variant ascertainment algorithm as previously described (*4*). The 3 ST468 strains differed from the ST452 reference strain by an average of 725 single-nucleotide polymorphisms (SNPs). When we plotted genome-wide SNPs of the ST468 strains against the genome of the reference strain (using BRIG [*11*]), we found a conspicuous area with an overabundance of SNPs (positions 210–320 kbp of the reference genome [Figure 1]). Nonrandom polymorphism distribution suggests recombination. To confirm this hypothesis, we analyzed the polymorphism data with BRAT NextGen (*12*), run with 20 iterations, 100 replicates, and a significance cutoff of 0.05. The analysis defined a region of recombination in strain PF-10 corresponding to positions 211,553–331,548 bp of the reference genome (Figure 1). A slightly narrower area of recombination was defined in strains PF-17 and PF-18 (211,553–323,601 and 249,415–323,601, respectively) (Figure 1). 

In all 3 strains, the gene *tkt*, used in the MLST scheme, was found within the region of recombination, which explained the MLST result that ST468 is a single-locus variant of ST452. The common region of recombination also contained genes involved in metabolic pathways, such as *nagA*, encoding an N-acetylglucosamine-6-phosphate deacetylase involved in sialic acid metabolism. The ability of certain streptococcal species to use sialic acid might play a role in the persistence and survival of these infecting organisms in vivo (*13*). Additionally, the region contained genes encoding the glycerol kinase GlpK, the aminopeptidase PepC, and the NAD synthetase nicotinamide adenine dinucleotide. This region also contains a bacteriocin (enterocin A) and penicillin-binding proteins PBP2X and PBP1A.

To identify the potential recombination donor, we first built a pseudoreference genome of strain PF-10. In brief, we performed de novo assembly of short reads generated for this strain as previously described (*5*), ordered obtained contigs against the reference genome of the ST452 strain NGBS572 with progressiveMauve (*14*), and concatenated the contigs with a separator that introduced a stop codon in every reading frame. Next, we aligned whole-genome sequence short reads of strains belonging to 12 different STs against the PF-10 pseudogenome and identified polymorphisms as described here. These 12 STs represent the 6 major CCs most frequently associated with GBS disease in humans (*9*). The pattern of polymorphisms strongly suggested that the donor of the recombined fragment was a CC17 strain (Technical Appendix Figure). Strains of CC17 are associated with neonatal infections, especially late-onset disease (*15*).

**Figure 2:**
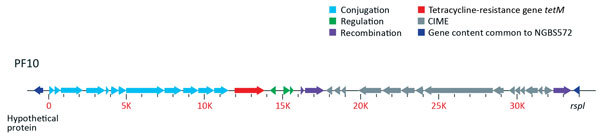
Representative diagram of a *tetM*-containing mobile genetic element (MGE) in group B *Streptococcus* sequence type (ST) 468 strains. Genome analysis discovered that the MGE is integrated at the 3′ end of gene *rspI*. Genes common between ST468 strains and the ST452 strain NGBS572 are shown in dark blue. Genes involved in conjugation are shown in light blue, regulatory genes in green, recombination genes in purple, and the gene encoding tetracycline resistance (*tetM*) in red. Cis integrative and mobilizable element genes (CIME) are shown in gray. Mbp, megabase pairs

Lateral exchange of antimicrobial drug resistance mediated by mobile genetic elements occurs frequently in GBS. The implications of these events (beyond the obvious gain of resistance to a particular antimicrobial agent) are not yet fully understood. For example, Da Cunha et al. recently presented convincing evidence that tetracycline resistance has contributed to the worldwide expansion of GBS clones causing disease in humans (*9*). Strains of one of the major STs associated with serotype IV GBS invasive disease, ST452, are sensitive to tetracycline (*5*). Our unpublished observations suggest that the genome of ST452 strains could be considered a hybrid genome combining sequences of ST23 and ST17. ST468 strains analyzed here have additional ST17 content and were resistant to tetracycline. We determined that the genomes of all 3 ST468 strains contained gene *tetM*, which was carried on a mobile genetic element (MGE) (Figure 2). Areas of this MGE have 99% identity (blastn, www.ncbi.nlm.nih.gov/blast/Blast.cgi) to transposon Tn*916*. The Tn*916*-like element lies upstream of cis-mobilizable genetic content. Further inspection revealed that the MGE is inserted in the 3′ end of gene *rspI* (positions 232,722–250,539 bp of the ST452 genome), which is a known hot spot for integrative conjugative element integration in GBS (*8*).

## Conclusions

WGS-based studies are expanding our understanding of genome reshaping in GBS and beginning to shed light on the population dynamics of this species. In particular, the use of WGS has shown that strains with multiple genome backgrounds are involved in the emergence of serotype IV GBS disease in North America (*5*,*6*). Here, we show that strains of one of these diverse backgrounds, ST468, have acquired additional genetic material from a neonatal-associated lineage by means of recombination. We also show that these isolates have acquired tetracycline resistance by lateral transfer. The changing epidemiology of GBS disease, with the involvement of a diverse array of strain genotypes, warrants continuing monitoring of colonization and invasive serotype IV GBS infections. Our data also support including this serotype in GBS vaccine formulations currently under development.

Technical Appendix Group B *Streptococcus* sequence type 468 strains acquired additional 120 kbp most likely from clonal complex 17 donor by homologous recombination.
