# Supplementary material for: Serotype IV Sequence Type 468 Group B Streptococcus Neonatal Invasive Disease, Minnesota, USA
Source: Emerg Infect Dis. 2016 Nov;22(11):1937–40. doi: 10.3201/eid2211.152031 (PMC5088005; doi:10.3201/eid2211.152031)
Supplement: Technical Appendix — Group B Streptococcus sequence type 468 strains acquired additional 120 kbp most likely from clonal complex 17 donor by homologous recombination. [file 15-2031-Techapp-s1.pdf]

Article DOI: http://dx.doi.org/10.3201/eid2211.152031

Serotype IV ST468 Group B *Streptococcus* Neonatal Invasive Disease, Minnesota, USA

Technical Appendix

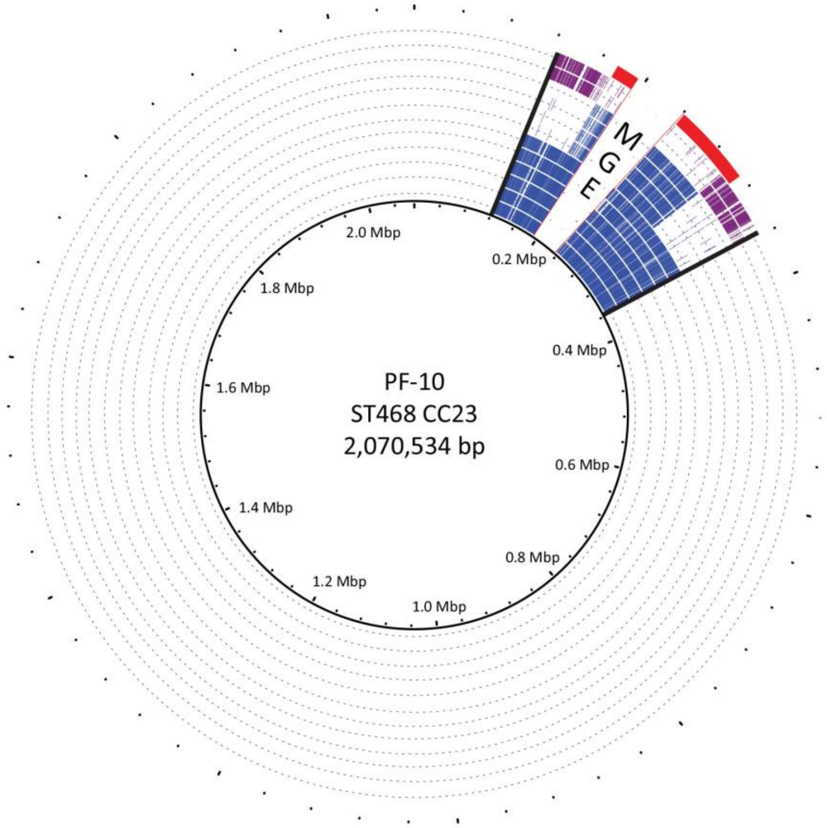

Technical Appendix Figure. Group B *Streptococcus* ST468 strains acquired additional 120 kbp, likely from CC17 donor by homologous recombination. Polymorphisms identified in strains of diverse sequence types relative to the pseudochromosome of strain PF-10 (representative of all 3 ST468 strains) are plotted for the area of recombination (highlighted in red in the outer ring). A minimal number of polymorphisms relative to PF10 were identified in this area for CC17 strains (ST17 and ST484, shown in purple), whereas for strains of all other STs tested, we identified >500 polymorphisms in this area. Conversely, when we analyzed regions extending 50 kbp upstream and downstream of the recombination area, we observed opposite results, with strains of CC23 (ST452, ST23, ST24, ST144) showing minimal number of polymorphisms. An MGE found within regions having undergone recombination is indicated by

MGE. The MGE was not considered in the recombination analysis. The ST of strains shown, from outermost ring to innermost is: ST17, ST484, ST452, ST23, ST24, ST144, ST459, ST26, ST3, ST110, ST10, ST1. CC, clonal complex; MGE, mobile genetic element; ST, sequence type.
